# Influence of suspended cenospheres on the mechanical characteristics and wear loss of natural fiber-reinforced hybrid composites: implications for biomedical applications and sustainable material management

**DOI:** 10.1039/d4ra06223j

**Published:** 2024-10-22

**Authors:** Bennehalli Basavaraju, Santhosh Nagaraja, Ashok R. Banagar, C. V. Srinivasa, B. T. Ramesh, Deden Ramdan, Muhammad Imam Ammarullah

**Affiliations:** a Faculty of Chemistry, MVJ College of Engineering Bangalore 560067 Karnataka India; b Department of Mechanical Engineering, MVJ College of Engineering Bangalore 560067 Karnataka India; c Faculty of Mechanical Engineering, PES Institute of Technology and Management Shivamogga 577204 Karnataka India; d Faculty of Mechanical Engineering, GM University Davanagere 577006 Karnataka India; e Faculty of Robotics and Automation, Symbiosis Institute of Technology (Symbiosis International University) Pune 412115 Maharashtra India; f Department of Management Science, Faculty of Social Science and Political Science, Universitas Pasundan Bandung 40261 West Java Indonesia deden.ramdan@unpas.ac.id; g Department of Mechanical Engineering, Faculty of Engineering, Universitas Diponegoro Semarang 50275 Central Java Indonesia imamammarullah@gmail.com; h Undip Biomechanics Engineering & Research Centre (UBM-ERC), Universitas Diponegoro Semarang 50275 Central Java Indonesia

## Abstract

The need for non-conventional natural fibres for synthesis of hybrid composites has gained momentum in the recent past. Taking into consideration this need, in the current study, hybrid composites were fabricated by reinforcing wood apple shell powder and coconut shell powder, in the resin with varying amounts of cenospheres (up to 20 wt% in increments of 5 wt%) to evaluate their mechanical and tribological properties. The densities of these composites were directly correlated with the quantity of additives utilized. Enhanced tensile and flexural properties were noted in composites containing 10 wt% cenospheres, along with 15 wt% wood apple shell powder and coconut shell powder, compared to other formulations. Dry sliding wear tests were performed at room temperature using a pin-on-disc apparatus, considering loading factors, travel distance, and speed. A hybrid composite consisting of 10 wt% cenospheres, subjected to a normal load of 10 N (1.02 kgf), and tested at a sliding speed of 1.5 m s^−1^ (90 m min^−1^) over a distance of 500 m, exhibited superior wear resistance compared to all other composite variations.

## Abstract 2

The need for non-conventional natural fibres for synthesis of hybrid composites has gained momentum in the recent past.

## Graphical abstract

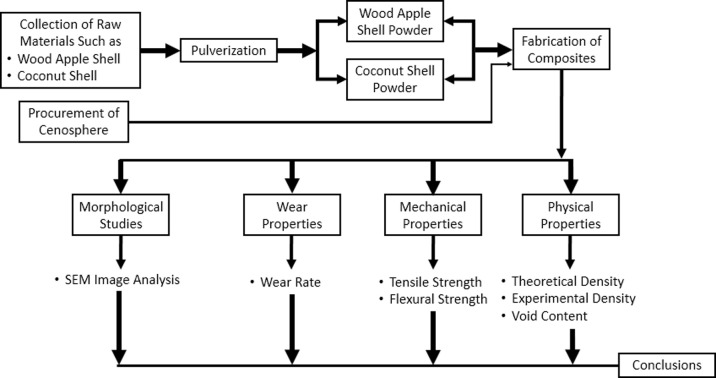

## 1. Introduction

The primary objective is to create composites that are cost-effective and environmentally friendly. Scientists and engineers have collaborated to develop economically attractive composites, leading to the adoption of innovative production methods in the composite sector.^1^ The proper disposal of composite materials, including ceramics, plastics, and synthetic fibers, has become a significant challenge for many companies in contemporary times due to stringent environmental laws and regulations. Several researchers have identified natural fibers and hybridization as effective solutions to address this issue.^2,3^

Despite the environmental benefits, natural fiber-reinforced polymer composites (NFRPCs) face challenges such as high-water absorption and suboptimal mechanical properties. These limitations can be mitigated by optimizing factors like fiber type, orientation, aspect ratio, and interface bonding. By chemical modification of the natural fibres, the physicochemical and mechanical properties of NFRPCs can be improved, resulting in greater bonding between the fibre surface and the polymer matrix.^4,5^

Natural fibers offer environmentally friendly and cost-effective alternatives to synthetic fibers due to their biodegradability, low density, and adequate mechanical properties, making them suitable for applications in industries such as automotive, aerospace, biomedical, and construction.^6,7^

Tribological performance of composites is influenced by factors such as material type, chemical treatment, polymer combinations, and manufacturing methods. Possible advantages include issues related to poor density, cost, and the availability of natural fillers.^8–10^ Certain researchers explored the wear behavior of polymer composites made by reinforcing different types of natural fibers and filler materials. Their focus was on investigating the feasibility of using nano-fillers with readily available plant fibers to produce wear-resistant hybrid composite materials. Moreover, the resulting composites demonstrated a notable decrease in friction coefficient and reduction in specific wear rate.^11–21^

Previous studies on jute fiber reinforced polylactide (PLA) have explored various chemical treatments and coupling agents to improve the mechanical and wear properties of natural fiber composites, highlighting the potential for enhancement through surface modification and matrix interaction.^22,23^ The thermal stability of jute-PP composites with melt-mixed MA-g-PP was cited as a reason for the improved wear resistance compared to composites with solution-treated jute fibers. A research was carried out on the wear characteristics of bamboo stem cross-sections under simulated soil conditions, using a combination of quartz sand, powdered bentonite, and water.^24^ The study found that the wear resistance of bamboo depended on the vascular fiber composition, with increased vascular fibers enhancing wear resistance. Faster sliding or larger abrasive particle sizes led to higher abrasion.

The study on the wear behavior of composites consisting of graphite-enhanced polyester and cotton revealed that the addition of cotton fibers to the polyester resin reduced the specific wear rate, and further reduction was observed with the addition of graphite.^25^ This study also revealed that adding cotton fibers improved the structural integrity of polyester resin under sliding wear conditions. Additionally, the coefficient of friction increased with the addition of cotton fiber, while it decreased with an increase in graphite content in the composite. An investigation on the wear and frictional characteristics of epoxy composites reinforced with modified betel nut fiber was conducted on a linear Tribo machine, subjecting the composites to sliding against grain sands.^26^ The results revealed instances of plastic deformation, pitting, and pull-out of betel nut fibers. When tested against coarse sand, the composite exhibited higher frictional coefficients. It was observed that abrasive wear in the composite is influenced by both sliding velocity and particle size, with higher sliding velocities correlating with increased weight loss. Another study explored the physico-mechanical and erosion properties of turmeric-spent (TS) and polypropylene (PP) green composites, examining their suitability for load-bearing and tribological applications.^27^ Various concentrations of TS (10%, 20%, 30%, and 40% w/w) were employed. The study compared the evaluated tensile strength of the composites to theoretically expected values using multiple theoretical models. The water absorption characteristics of the composites were also identified. Notably, the inclusion of TS filler was found to decrease the abrasion resistance of PP/TS composites.

Hybridization techniques aim to enhance mechanical properties in composite material systems. This involves incorporating supplementary reinforcements like whiskers or fillers, such as rigid spherical fillers, typically ceramic or metallic, to improve surface quality and strength. Cenospheres, hollow micro spherical alumino-silicate particles found in fly ash, offer a cost-effective alternative due to their industrial waste status. Their fine dispersion, homogeneity, inertness, and chemical stability make them valuable fillers in polymer matrix composites.^28^ Cenospheres, tiny hollow spheres derived from fly ash, have emerged as a notable solution for erosion-related challenges owing to their distinct properties and potential advantages.^29,30^ Erosion, a consequence of solid particle impacts on surfaces, presents issues such as material deterioration, increased maintenance expenses, and diminished operational efficiency across various sectors like oil and gas, mining, and transportation.^31^ Integration of cenospheres into erosion-resistant materials offers a promising avenue to mitigate erosion-induced damage, thereby enhancing the durability and effectiveness of critical components.^32^ Cenospheres are added to polymer matrices to reduce material consumption, improve composite properties, and lower costs. These benefits extend to various applications, including low-weight cementitious composites for superior thermal insulation properties and specific strength values.^33^ Studies have explored the incorporation of cenospheres into fiber reinforced cement composites, aramid fiber reinforced phenolic polymer matrix composites, and ceramic/phenolic composites, demonstrating improved properties and cost-effectiveness.^34–36^ The appeal of cenospheres in erosion applications stems from their lightweight nature and exceptional mechanical attributes.^37^ With densities typically falling between 0.4 to 0.8 g cm^−3^, cenospheres substantially reduce the weight of composite materials while preserving structural integrity. This weight reduction proves advantageous in environments prone to erosion, where lighter materials can mitigate impact forces and minimize surface wear.^38^ Incorporating cenospheres into matrices such as polymers, ceramics, or metals enhances material hardness, toughness, and resistance to impact and abrasion. The hollow structure of cenospheres acts as a cushion, absorbing and dissipating energy from particle impacts, thus reducing erosion rates and safeguarding underlying materials.^39–41^

Moreover, the chemical composition of cenospheres contributes to their resilience against erosion. Mainly composed of silica, alumina, and other oxides, cenospheres exhibit high resistance to chemical attack from acids, alkalis, and corrosive substances. This chemical durability makes cenosphere-reinforced materials suitable for erosion applications in harsh environments where chemical exposure is a concern. The spherical shape of cenospheres further enhances their erosion resistance. Their smooth, spherical surface minimizes the contact area between particles and materials, thereby reducing frictional forces and erosive wear. This property helps maintain material integrity and prolong service life in erosive environments.^42,43^

In recent years, bio-ceramics derived from sustainable resources have garnered significant attention. Various approaches have been explored, including the use of natural, non-toxic biomaterials, and the recycling and reuse of waste materials. Notably, cenosphere, a byproduct of fly ash produced during the pulverized coal combustion, has been increasingly recognized for its potential as a sustainable ceramic material in biomedical engineering. Due to its inherent properties, such as low specific density, high hardness, and chemical inertness, cenosphere has been extensively utilized as a filler material in the fabrication of composites for bone. This highlights the promising potential of cenosphere in biomedical engineering.^44–46^ However, to the best of our knowledge, there is a lack of studies exploring the potential of cenosphere for direct applications, such as a ceramic bio-scaffold.

The growing demand for cementitious materials with reduced density and improved thermal properties has led to the development of lightweight alternatives. These lightweight cementitious materials offer advantages such as lower weight, better thermal performance, and cost-effectiveness compared to traditional cementitious materials. One method to produce these lightweight materials is by incorporating inexpensive, waste-derived lightweight aggregates or fillers, which help reduce the dead load of the material. Additionally, utilizing waste materials in cementitious products provides an effective way to mitigate the negative environmental impacts of improper waste disposal. Cenospheres, in particular, are a promising waste material that can be recycled into cementitious products to create lightweight materials. Their inclusion has also been shown to enhance workability and insulation properties.^47,48^

It's important to note that cenospheres have applications beyond cementitious materials. They can be utilized in aeronautical materials, plastics, rubber, as well as heat and sound insulators. Despite the many promising benefits of using cenospheres, one significant drawback is their high cost. However, with the anticipated rapid growth and large-scale commercial applications of cenospheres in the coming years, it's expected that their cost will decrease in the near future.^47,49,50^

In summary, the development of cost-effective and environmentally sustainable composite materials is important in addressing the challenges posed by traditional synthetic materials. The exploration of natural fiber-reinforced composites, despite their inherent limitations, presents a promising avenue for creating materials with improved mechanical properties and reduced environmental impact. The integration of advanced fillers like cenospheres offers the potential to overcome these limitations, enhancing the performance of composites in various industrial applications. This research seeks to leverage the unique properties of cenospheres, combined with natural fibers, to develop composites that are not only lightweight and cost-effective but also possess superior wear resistance and mechanical strength. The outcomes of this study could contribute significantly to the advancement of composite materials, offering viable alternatives for industries ranging from construction to biomedical engineering.

## 2. Materials and methods

### 2.1 Epoxy (matrix) and hardener

Epoxy of diglycidyl ether of bisphenol A type (trade name LAPOX L-12) and triethylenetetramine (trade name K-6) hardener were purchased from Imox Chemicals from Bengaluru, India (specification obtained from technical data sheet by supplier is given in Table 1). Resin to hardener ratio was 10 : 1 and the pot life of mixed resin was 30–40 min. After considering the adoptability and applicability, epoxy resin is selected for composite fabrication due to its outstanding mechanical properties, high corrosion and moisture resistance, ease of manufacturing, exceptional tensile and compressive strength, minimal shrinkage during curing, and versatile bonding capabilities with various fibers.

**Table 1:** Specification of LAPOX L-12 and hardnener

Property	Unit	Value
**LAPOX L-12**		
Epoxide equivalent	g eq^−1^	182–192
Epoxy value	eq kg^−1^	5.2–5.5
Viscosity at 25 °C	mPa s	9000–12000
Hardener K-6		
Refractive index at 25 °C		1.4940–1.5000
Water content		1% max

**Properties of cured resin without reinforcement**		
Tensile strength	N mm^−2^	50–60
Compressive strength	N mm^−2^	110–120
Flexural strength	N mm^−2^	130–150
Impact strength	kJ m^−2^	17–20
Modulus of elasticity	N mm^−2^	4400–4600

**Scheme 1:**
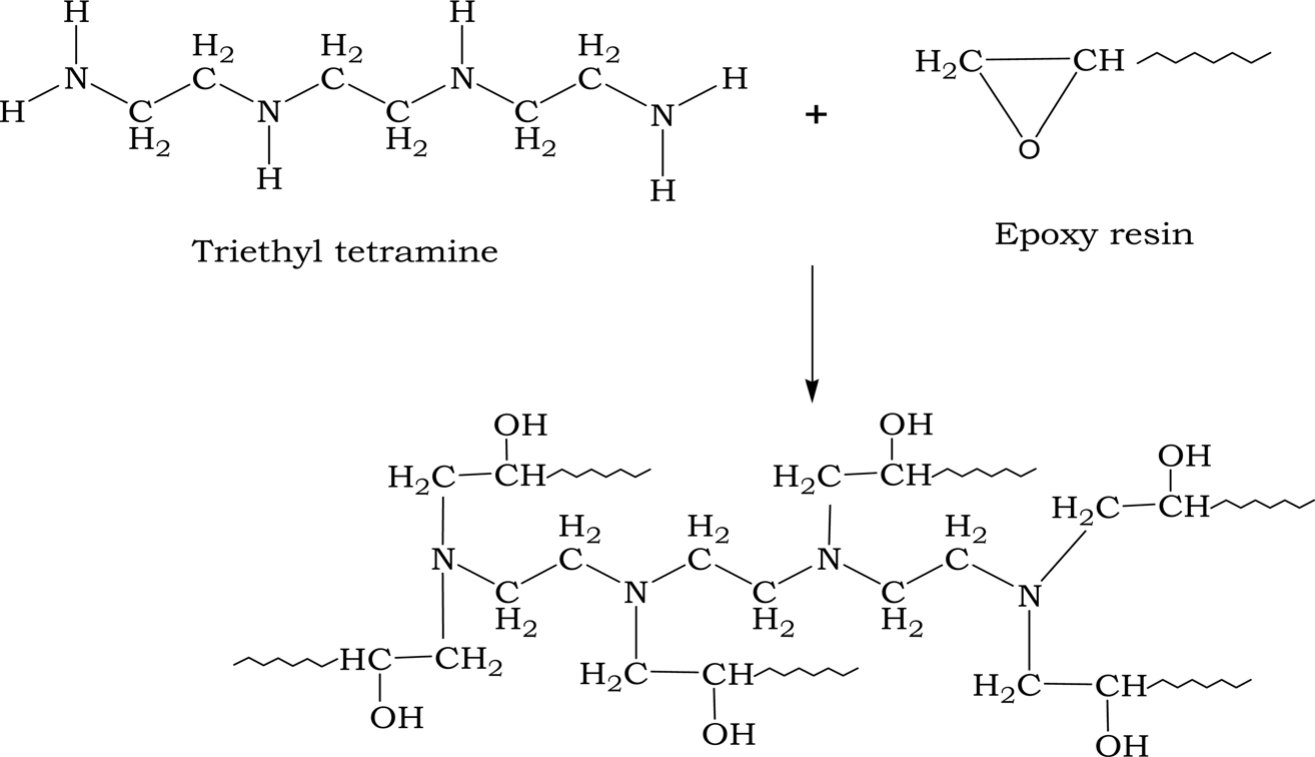
Interaction between triethylenetetramine and epoxy resin.

Chemical bonding is based on reactive chemical groups in the reinforcement and matrix, which defines the strength of formed interface. Epoxy monomers have active oxirane functional groups because of their high molecular strain. This group tends to open its ring for nucleophiles such as OH^−^. Initially, epoxy reacts with a hardener to form a product free from epoxy groups (Scheme 1), where adhesive properties are improved, and mechanical adhesion is possible along the reinforcement. The strength of the reinforcement-to-epoxy matrix bond is a significant factor for obtaining good characteristics in a composite material.

### 2.2 Composites made with powdered CNS, WAS, and cenosphere

**Fig. 1:**
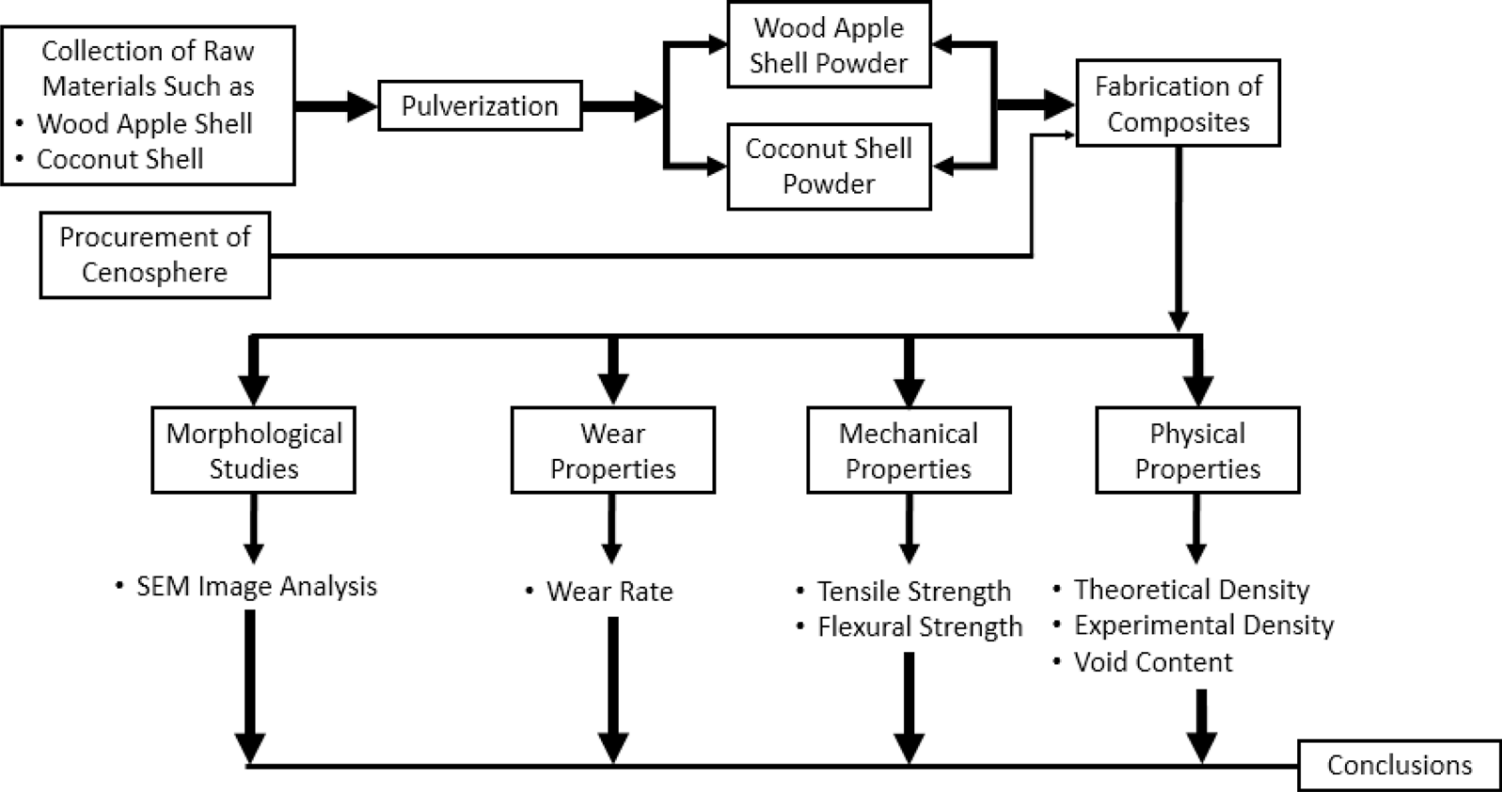
Methodology adopted in the present work.

**Fig. 2:**
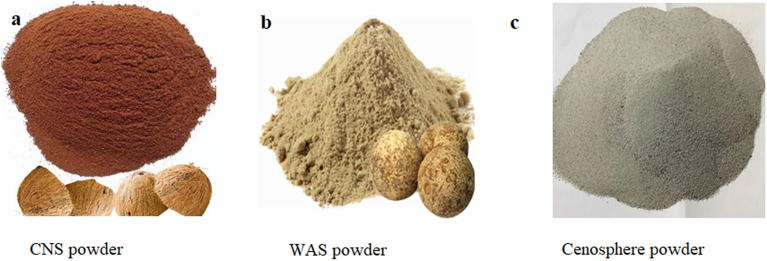
Ingredients (powder form) used in the present work: (a) CNS powder, (b) WAS powder, and (c) cenosphere powder.

The methodology adopted in the current study is illustrated in Fig. 1, showcasing the approach adopted in the current work to examine natural fibers reinforced hybrid composites. Coconut is abundantly available in Asia and its aged fruit serves various purposes. However, the coconut shell is often discarded or burned. In the current process, coconut shells (CNS) are ground into a fine powder (Fig. 2(a)) after complete drying. Similarly, wood apple shell (WAS) (Fig. 2(b)) is another material utilized in natural composite fabrication, prepared in a manner similar to CNS. Cenosphere powder (Fig. 2(c)) is sourced from Manjunath traders in Mandya.

To create the resin mixture, 70% of the resin (in a 10 : 1 weight ratio) is thoroughly blended with the hardener after 15 minutes of mechanical mixing at 50 °C. The hardener is gradually introduced to prevent rapid chemical reactions. Five different hybrid composites were prepared by reinforcing a mixture of CNS, WAS, and cenosphere powders into epoxy resin. The proportions of the reinforcing agents, CNS and WAS, were fixed at 15 wt%, while the cenosphere powder varied from 0 to 20 wt% in 5% increments. The fabrication process began by blending the fixed amounts of CNS and WAS powders with the resin mixture at a rotor speed of 60 rpm. Subsequently, the specified percentage of cenosphere powder was added to form the hybrid composites. This mixture was then carefully placed into a square steel mold and cured at room temperature under a 25 kg load for one day, ensuring the formation of well-bonded and uniform composite structures. Teflon sheets are applied to the mold edges to prevent sticking. The resulting composites were cut into specimens with the required dimensions in accordance with ASTM standards for the various trials. Table 2 outlines the composite terminology and configuration differences from previous studies.

**Table 2:** Details of composite sample compositions

Nomenclature	% CNS (wt%)	Mass of CNS (g)	% WAS (wt%)	Mass of WAS (g)	% Epoxy with hardener (wt%)	Mass of cenosphere (wt%)
CWC-15:0	15	70.5	15	44.8	70	0
CWC-15:5	15	70.5	15	44.8	65	5
CWC-15:10	15	70.5	15	44.8	60	10
CWC-15:15	15	70.5	15	44.8	55	15
CWC-15:20	15	70.5	15	44.8	50	20

### 2.3 Testing methodology

The physicochemical attributes of the resulting composites, such as density and void content, as well as mechanical properties like tensile and flexural (bending) strength, along with sliding wear parameters, were systematically investigated.^51^ The law of mixing was employed to calculate the theoretical density, while the Archimedes standard was applied to determine the observed densities. The void percentage of the composite was computed by normalizing the disparity between the experimental and theoretical densities.

Tensile tests were conducted according to the ASTM D-638-14 standard using a ball screw driven UTM (TEC-SOL TTM 600C) equipped with a 20 kN load cell. Based on the testing standard, specimens were prepared with a dimension of 165 mm length, 19 mm width and 3.2 mm thickness for tensile tests. The crosshead speed of the machine was kept constant at 5 mm min^−1^ during the test. A total of five specimens of each type of composite sample was tested and their mean value was calculated.

**Fig. 3:**
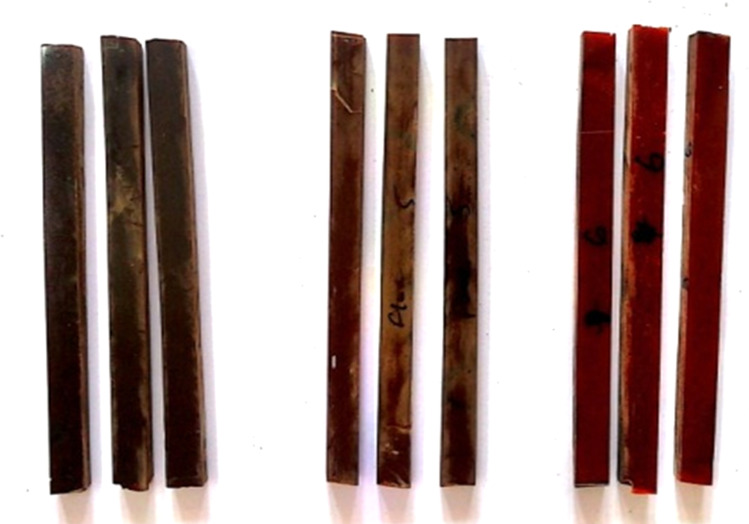
Fabricated samples for mechanical characterization.

Three-points bending flexural test was conducted using the same universal testing machine (UTM). As D-790-03 testing standard was followed, the dimension of the specimen was 125 mm × 12.7 mm × 3.2 mm. The crosshead speed was 5 mm min^−1^ and minimum five specimens of each sample type were tested. Fig. 3 illustrates a subset of the sample specimens created for mechanical characterization.

A micromatic Technologies made High Temperature Tribometer (Wear Testing Machine) is specifically the pin-on-disc machine aligned with ASTM D4060-10, was utilized to assess the wear characteristics of the composites during dry sliding. The standard benchmark for evaluating abrasion resistance involved measuring weight loss after a specified number of abrasion cycles. Two rotating abrasive wheels, each measuring 50 mm in diameter and 12.6 mm in thickness, applied an impact force of 1000 g to the test materials. The motor operated at a speed of 60 rpm for approximately 10 000 cycles.

The fractured surfaces of the samples were analyzed with the help of a Scanning Electron Microscopy (SEM). The prepared composite specimens were observed by SEM (Hitachi S-3400 N) at different magnifications. The specimen surfaces are coated with silver by ion sputtering at an accelerating voltage of 15 kV.

## 3. Results and discussions

### 3.1 Physical properties

While developing a lighter weight structural composite, the density of the composite is the most essential aspect to consider. Density is typically influenced by the relative percentages of strengthening and matrix materials. Using the eqn (1), the theoretical density (*ρ*_th_) of hybrid composites were determined in the context of the weight percentage of the various constituents.1
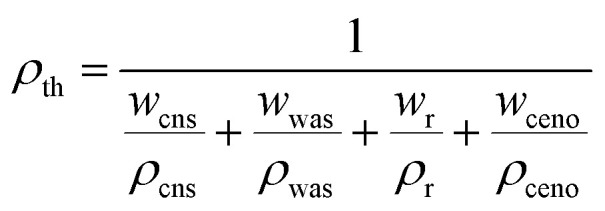
where ‘*w*’ is weight, ‘*ρ*’ is density, ‘cns’ is coconut shell, ‘was’ is wood apple shell, ‘*r*’ is resin and ‘ceno’ is cenosphere. Using eqn (2), the percentage of void content (*V*_c_) was calculated from the theoretical and experimental densities of the composites. Where *ρ*_th_ represents the theoretical density and *ρ*_ex_ represents the experimental density.2
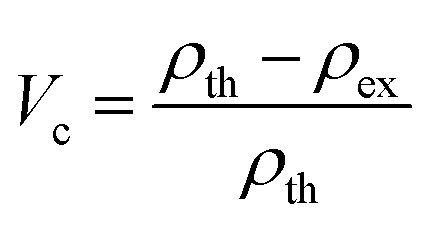


**Fig. 4:**
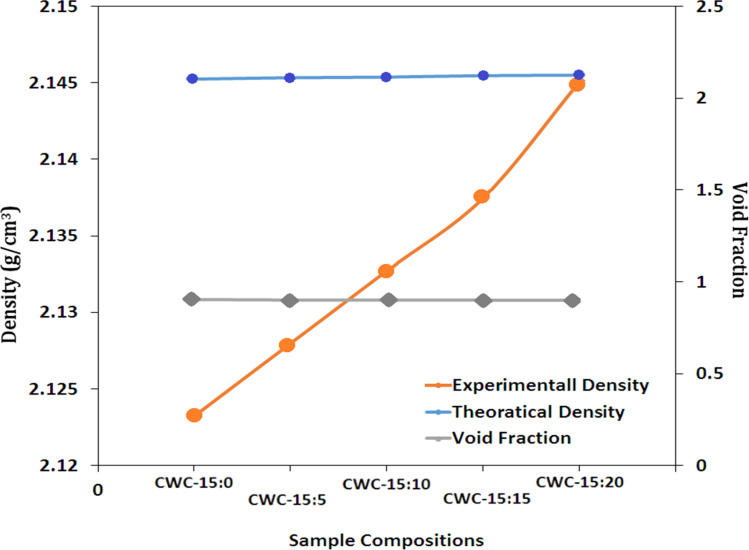
Representation of densities and void contents of the hybrid bio-composites prepared.

Fig. 4 presents a summary of the theoretical densities, experimental outcomes, and corresponding void percentages of the composites, highlighting the influence of heliosphere reinforcement. The findings illustrate a significant impact of cenosphere content on both experimental and theoretical densities of the resulting composites, ranging from 2.1233 to 2.1451 g mm^−3^ and 2.1024 to 2.1261 g mm^−3^, respectively. In the case of low-density epoxy composites, the addition of cenosphere led to an increase in composite density. Moreover, as the cenosphere concentration increased, there was a marginal reduction in the void percentage. It is noteworthy that the average particle size of coconut shell and wood apple shell were 300 μm and 280 μm respectively. The reduction in void content observed in hybrid composites is directly correlated with an escalation in the percentage of cenospheres. This increase in cenosphere proportion up to 10% contributes to the enhancement of mechanical properties in the prepared hybrid composites. Specifically, the CWC (cenosphere + wood apple shell powder + coconut shell powder) composite demonstrates superior fiber/matrix interface bonding, manifested by a noticeable decrease in voids. The formation of voids in polymer composites can be ascribed to processing effects originating from various sources, such as entrapment of air bubbles within the epoxy matrix, presence of residual solvents, and release of volatiles during resin curing.^39^ These voids may manifest at the fiber/matrix interface or within the fiber lumens, exerting an impact on composite properties and resulting in a reduction in composite density.^52^ Particulate natural fibers exhibit improved wettability with epoxy resin, leading to the noteworthy observation that CWC hybrid composites contain significantly fewer voids.^53^ This reduction in voids can be attributed not only to the inherent characteristics of natural fibers in particulate form but also to the incorporation of cenospheres during the preparation of CWC composites. The presence of high voids in composites can detrimentally affect fatigue resistance, increase susceptibility to water absorption, and introduce greater variability in mechanical properties.

### 3.2 Mechanical properties

**Fig. 5:**
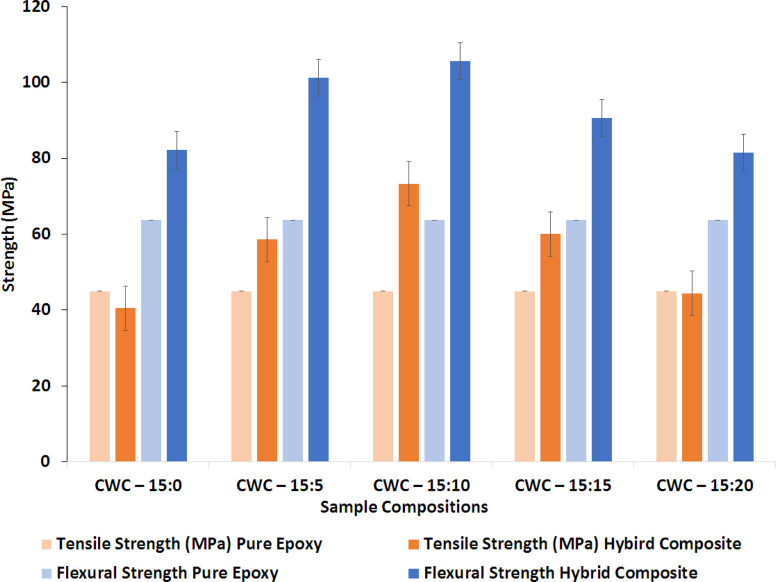
Tensile and flexural strength ratings for hybrid bio-composites and produced pure epoxy samples.

Fig. 5 illustrates the tensile and flexural strengths of each composite sample, along with the corresponding cenosphere content. The arrows indicate the standard deviation in the dataset. Among the various hybrid composites reinforced with coconut shell powder (CNS), wood apple shell powder (WAS), and cenosphere, the CWC-15:10 hybrid composite (with 10% cenosphere) exhibits superior tensile strength compared to the CWC-15:0, CWC-15:5, CWC-15:15, and CWC-15:20 composites. This improvement is attributed to the reduced void content and enhanced filler dispersion, which promotes better matrix-filler bonding. However, as the cenosphere content increases beyond 10%, there is a tendency for cenosphere localization, leading to the formation of fractures. These fractures propagate under increased loading, resulting in composite failure under lower stress conditions.

Similarly, the flexural strength of the composites improved with increasing cenosphere content, reaching a peak of 105.63 MPa at 10 wt% cenosphere. This enhancement highlights the effectiveness of cenosphere as a stress distributor and crack deflector at optimal levels. However, further increases in cenosphere content (such as 15% and 20%) led to a decline in flexural strength, similar to previous studies on cenosphere-reinforced composites. This reduction can be linked to the agglomeration of cenospheres, which creates localized stress concentrations and reduces the composite's overall strength.^39^

The formation of voids in polymer composites is influenced by several factors, including insufficient resin infusion, trapped air, moisture volatilization, low-molecular-weight components, and uneven filler distribution. The CWC composite, which includes cenosphere, wood apple shell powder, and coconut shell powder, successfully addresses these issues by enhancing the fiber/matrix interface bonding. Cenospheres, being hollow and lightweight, lower the composite's density while filling gaps, thus reducing void concentration. Natural fillers such as wood apple shell powder and coconut shell powder also improve mechanical interlocking at the fiber/matrix interface, and their porous nature allows for better resin infiltration, minimizing void formation. This combination enhances matrix wettability and promotes a more uniform resin distribution with fewer voids. The synergistic effect of these fillers improves interfacial bonding, facilitating stress transfer and further reducing the potential for void formation. As a result, the unique blend of fillers in the CWC composite plays a critical role in minimizing void formation and enhancing the overall mechanical performance.

The optimal cenosphere content for the composite is 10%, which significantly improves both tensile and flexural strengths. At this concentration, cenospheres act as effective stress distributors and crack deflectors, with their hollow structure aiding in the even distribution of stress across the composite matrix. This slows down the initiation and growth of cracks, thus enhancing mechanical performance. Additionally, the balanced interaction between the matrix and filler at this level allows for efficient load transfer, further strengthening the composite. However, when the cenosphere content exceeds 10%, mechanical properties and wear resistance begin to deteriorate. This decline is due to increased porosity, as the hollow cenospheres create more voids, weakening the matrix-filler interface. Furthermore, excessive cenosphere content leads to particle clustering, which acts as a crack initiation site and compromises the composite's integrity. The reduced volume of matrix material also contributes to this degradation, as the matrix is essential for binding the composite. Therefore, while 10% cenosphere content optimizes tensile and flexural properties, higher concentrations result in reduced mechanical performance due to porosity, agglomeration, and insufficient matrix material.

Although void content plays a significant role in influencing the mechanical properties of composites, it is not the only factor. SEM imaging provides valuable insights into the microstructural changes that affect tensile and flexural strengths. The SEM images (Fig. 7) highlight several critical aspects, including matrix-filler bonding, filler dispersion, and fracture mechanisms. The degree of bonding between the matrix and fillers, such as cenosphere, wood apple shell powder, and coconut shell powder, is crucial for enhancing tensile and flexural strength. A stronger matrix-filler interface promotes better load transfer, improving mechanical properties regardless of void content. Additionally, the uniform distribution of fillers observed in the SEM images leads to more effective stress distribution, reducing weak points caused by agglomeration. Finally, the SEM images of the fractured surfaces reveal failure mechanisms, such as fiber pull-out, crack propagation, and micro-void formation, which are essential for understanding the composite's tensile and flexural behavior and explaining the variations in mechanical properties.

In summary, the optimal cenosphere content of 10% significantly improves the tensile and flexural properties of the CWC composite by enhancing stress distribution and filler–matrix interactions. Exceeding this concentration leads to the development of voids, agglomeration, and weaker matrix bonding, ultimately reducing the composite's mechanical performance.

### 3.3 Abrasion/wear properties

**Fig. 6:**
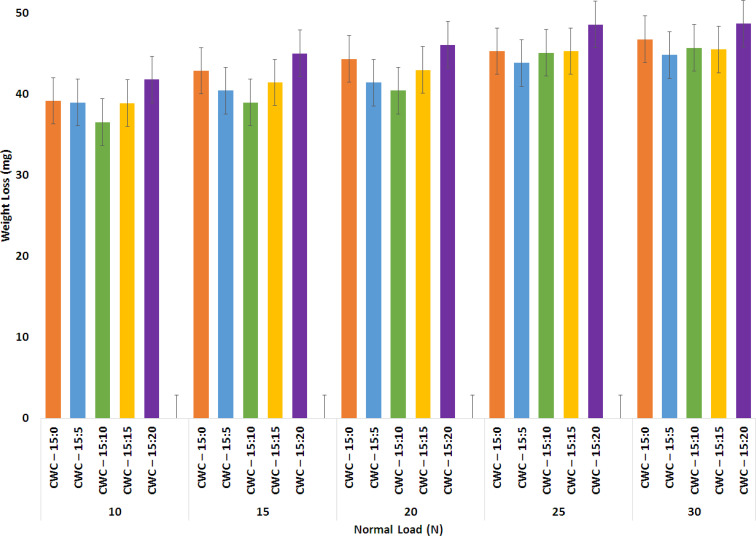
Wear rate for the various hybrid composites prepared in the present work.

Based on the weight loss observed in Fig. 6, the wear resistance of the hybrid composite specimens was assessed. This characteristic aligns with their mechanical properties concerning surface features. A comparison reveals that specimens subjected to higher loads experience greater weight loss compared to those with lower loads as a natural tendency of wear loss as weight increases. Furthermore, an increase in cenosphere content (up to 10 wt%) in the CWC hybrid composite samples results in a reduction in the wear rate for the bio-composites. However, a further increase in cenosphere percentage leads to a decreased wear rate due to the weakened inter-bonding among the constituents of the bio-composites.

The wear rate increased as the cenosphere percentage rose from 15% to 20%. This indicates an incongruity between the bio-composites' capacity to withstand greater weight, evidenced by a load increase from 25 N to 30 N during the wear test, and the escalating cenosphere content.

### 3.4 Morphology

SEM imaging was used to analyse the microstructural features of the composite specimens. The fractured surfaces displayed noticeable pores, some as large as several hundred microns, which indicate the presence of air voids and cenosphere particles within the material. These pores likely resulted from air trapped during fabrication, either due to inadequate resin infusion or air entrapment during mixing and moulding. Such voids serve as weak points in the structure, potentially leading to premature failure under stress and contributing to the overall porosity observed in the SEM images.

Cenosphere particles, being hollow and lightweight, are expected to reduce the composite's density and enhance its mechanical properties by acting as fillers. However, if not uniformly distributed or bonded properly to the matrix, they can create larger pores or voids. The interaction between the cenospheres and the matrix during fracture provides insights into their bonding. Well-bonded cenospheres can help deflect cracks and absorb energy, thus improving the toughness of the composite. However, large pores visible in the images suggest that in some areas, cenospheres did not bond well with the matrix, resulting in debonding or pulling out during fracture. This inadequate bonding can cause stress concentration points, eventually leading to crack formation.

**Fig. 7:**
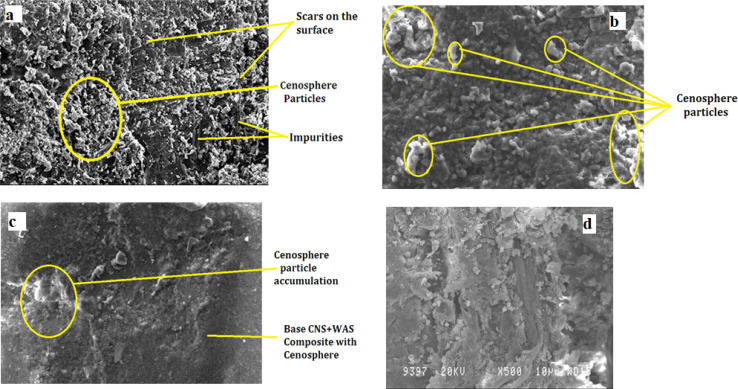
SEM images showing the effect of cenosphere distribution on fracture surfaces: (a) scars/scales on the hybrid composite (CWC 15:10) surface; (b) plain surface of the hybrid composite (CWC 15:10); (c) worn-out surface of the hybrid composite (CWC 15:10) and SEM images of composites (CWC 15:10) after the wear tests.

Fig. 7(a) shows SEM images of the hybrid composite (X500, 50 μm), revealing surface imperfections and the presence of cenosphere particles. The roughness observed in these images highlights areas where wear may have occurred, providing insights into how the material behaves under stress. In Fig. 7(b), cenosphere particles (X500, 10 μm) are seen evenly distributed across the surface of the composite, indicating uniform dispersion. This uniform distribution reduced the presence of pores and pits in the material, contributing to better mechanical performance.

Fig. 7(c) reveals a partial reaction between the cenosphere and the matrix, where some cenospheres appear partially consumed or broken, indicating interaction during the fracture process. However, as the cenosphere content increases, so does the overall porosity of the composite. This increased porosity, along with irregular pore shapes, can be attributed to localized accumulation of cenospheres, particularly in composites with higher weight fractions.

Fig. 7(d) illustrates the fractured surface, emphasizing the brittleness of the composite due to the incorporation of cenospheres. The image shows how cenospheres, along with wood apple shell (WAS) and coconut shell (CNS) particles, are distributed within the bio-composite matrix. Several visible pores suggest the potential for micro-cracks, which could lead to failure under high load conditions. The waxy nature of natural fiber reinforcements, combined with cenosphere particles, may cause composite agglomeration at elevated temperatures and speeds, leading to increased wear. However, the addition of cenosphere particles helps limit the accumulation of natural fiber reinforcements, reducing wear by minimizing interactions between WAS and CNS particles.

Overall, the SEM images demonstrate the critical role of processing conditions in reducing air voids and enhancing the bonding between cenospheres and the matrix. By optimizing these conditions, the porosity can be minimized, leading to improved mechanical interlocking and better overall performance of the composite.

To summarize, polymers and polymer-based composites are utilized as matrices or degradable carriers within the human body, as well as construction materials. In the first application, they function as carriers for drugs that are released at inflammation sites or as part of targeted treatments. The use of cenospheres in biomedical engineering and material management shows great promise, particularly in creating composite materials and standalone applications. In dental prosthetics and epitheses, cenospheres can be combined with ceramics and polymers to develop materials that offer high mechanical strength while being easy to use during treatment. This could address the current controversy surrounding zirconium oxide in dentistry due to its phase instability. Additionally, cenospheres could be used to enhance carbon fibers, leading to the production of ceramic-carbon materials for medical purposes, and to develop metal-ceramic composites for lighter orthopaedic implants, potentially enriched with drugs to reduce inflammation and postoperative complications.^54,55^

Research also highlights the potential of cenosphere-reinforced natural fiber composites in biomedical applications. The enhanced tensile and flexural strength provided by cenosphere makes these composites suitable for durable and flexible implantable materials, such as bone plates or joint prostheses. Their improved wear characteristics at optimal cenosphere levels could extend the lifespan of load-bearing implants like hip or knee replacements.^56^ SEM analysis of filler dispersion and void content further suggests that these composites could be useful in tissue engineering scaffolds, where controlled porosity and mechanical properties are critical for effective tissue regeneration. Moreover, these composites show potential for developing flexible biomedical devices that require a balance of strength and adaptability, such as stents or wearable sensors.^57,58^

In the second case, the incorporation of cenospheres, a by-product of coal combustion, into these composites aligns with sustainable practices by reducing waste and lowering material costs. Optimizing cenosphere content allows manufacturers to create stronger, lighter, and more durable building materials, which could replace traditional materials like wood or concrete in applications such as partitioning boards, flooring, and prefabricated buildings. This not only enhances the performance and longevity of construction materials but also promotes more sustainable construction practices by reducing the need for frequent replacements and improving lifecycle management.

## 4. Conclusions

In general, increasing particulate fibers in composites tends to reduce their strength. However, the incorporation of cenosphere led to improvements in both tensile and flexural strength, with this enhancement being most effective at a 10% addition of cenosphere to the composites. Beyond this level, further increases in cenosphere content caused localization, leading to a decline in mechanical properties. Wear studies also showed that minimal cenosphere reinforcement improved wear characteristics. However, as cenosphere content increased, the composites became more brittle, which negatively affected wear performance. The optimal range of 5–10% cenosphere in the CNS + WAS hybrid composites resulted in slight improvements in wear characteristics across all combinations in this study.

These composites show great potential for biomedical applications, such as bone plates and joint prostheses, which require materials with high mechanical strength and durability to withstand physiological loads. For load-bearing implants like hip or knee replacements, wear resistance is crucial to prevent implant failure and extend the lifespan of the device. In tissue engineering, these composites’-controlled porosities, as indicated by SEM analysis, allows for proper cell migration, nutrient diffusion, and waste removal, all of which are essential for tissue regeneration. The uniform microstructure resulting from well-dispersed cenosphere and reduced void content further enhances the mechanical integrity and biocompatibility of the scaffolds.

Additionally, many biomedical devices, such as stents and wearable sensors, require materials that are both strong and flexible to adapt to the dynamic environment of the human body. The balanced mechanical properties achieved through cenosphere reinforcement make these composites well-suited for such applications, where both flexibility and mechanical integrity are critical. Beyond biomedical uses, the hybrid composites developed in this study also hold promise for construction applications, such as partition boards, walls, flooring, window and door frames, roofing materials, and mobile or prefabricated buildings.

In conclusion, the study highlights that carefully controlling filler content, along with ensuring strong matrix-filler interactions, can result in hybrid composites with superior mechanical properties. Proper optimization of these factors allows for the development of versatile materials with potential applications in both biomedical and construction fields.

## Data availability

The necessary data used in the manuscript are already present in the manuscript.

## Author contributions

Basavaraju B.: conceptualization, data curation, formal analysis, funding acquisition, investigation, methodology, project administration, resources, software, supervision, validation, visualization, writing – original draft, writing – review & editing. Ramesh B. T.: conceptualization, data curation, formal analysis, funding acquisition, investigation, methodology, project administration, resources, software, supervision, validation, visualization, writing – original draft, writing – review & editing. Ashok R. Banagar: conceptualization, data curation, formal analysis, funding acquisition, investigation, methodology, project administration, resources, software, supervision, validation, visualization, writing – original draft, writing – review & editing. Srinivasa C. V.: conceptualization, data curation, formal analysis, funding acquisition, investigation, methodology, project administration, resources, software, supervision, validation, visualization, writing – original draft, writing – review & editing. Santhosh Nagaraja: conceptualization, data curation, formal analysis, funding acquisition, investigation, methodology, project administration, resources, software, supervision, validation, visualization, writing – original draft, writing – review & editing. Deden Ramdan, conceptualization, data curation, formal analysis, funding acquisition, investigation, methodology, project administration, resources, software, supervision, validation, visualization, writing – original draft, writing – review & editing. Imam Ammarullah: conceptualization, data curation, formal analysis, funding acquisition, investigation, methodology, project administration, resources, software, supervision, validation, visualization, writing – original draft, writing – review & editing. The authors listed have significantly contributed to the development and the writing of this article.

## Conflicts of interest

The authors declare no conflict of interest.
